# Dishing out mini-brains: Current progress and future prospects in brain organoid research

**DOI:** 10.1016/j.ydbio.2016.06.037

**Published:** 2016-12-15

**Authors:** Iva Kelava, Madeline A. Lancaster

**Affiliations:** MRC Laboratory of Molecular Biology, Cambridge Biomedical Campus, Francis Crick Avenue, CB2 0QH Cambridge, United Kingdom

**Keywords:** Organoid, Cortex, Human brain development, *In vitro*, Neurological disorder, Neural differentiation, Stem cells

## Abstract

The ability to model human brain development *in vitro* represents an important step in our study of developmental processes and neurological disorders. Protocols that utilize human embryonic and induced pluripotent stem cells can now generate organoids which faithfully recapitulate, on a cell-biological and gene expression level, the early period of human embryonic and fetal brain development. In combination with novel gene editing tools, such as CRISPR, these methods represent an unprecedented model system in the field of mammalian neural development. In this review, we focus on the similarities of current organoid methods to *in vivo* brain development, discuss their limitations and potential improvements, and explore the future venues of brain organoid research.

## Highlights

• Brain organoids currently model early human embryonic and fetal brain development to a remarkably high degree.
• Brain organoids successfully model several neurodevelopmental conditions – microcephaly, idiopathic autism, Zika infection.
• Modeling later neurodevelopmental events will require improvements to nutrient supply and sustained culture conditions.

## 1 Introduction

The human brain is one of the most complex organs in the animal kingdom, both structurally and functionally. These characteristics have captured scientists' interest for many years. However, not only does the brain's complexity present a challenge for the study of this unique organ, but also its inaccessibility for experimental manipulation. Therefore, researchers have traditionally utilized animal models for the study of adult and embryonic brain development. These studies have provided the foundation for our current understanding of brain development and function. Nonetheless, understanding of human brain development has been limited to those features that are shared among mammals and other vertebrates. Thus, the study of human or primate specific features of the brain have been limited to observations in post-mortem tissue, and functional studies have, until very recently, been impossible.

In order to perform functional studies in human brain development, researchers were in the need of an *in vitro* system that would recapitulate the features of the developing brain *in vivo*. Just a decade ago, this was thought to be unattainable. But numerous studies with tissues and cells cultured *ex vivo*, and the advent of pluripotent stem cells, have paved the way for three-dimensional models of developing neural tissue that model the developing brain with remarkable fidelity. The development of such 3D *in* vitro cultures, in which cells self organize into complex structures, has recently brought into usage the term “organoid”, previously an imprecisely defined term used for many different structures. Currently, in the context of *in vitro* cultures, there are ongoing debates as to at which level of complexity an embryoid body becomes an organoid. In this review, we will use the term organoid for 3D cultures which replicate not only the complexity of the cell types present in the organ, and the processes of self-organization of the tissue, but also the main organization of the whole organ, in this context the appearance of different brain regions (Lancaster and Knoblich, 2014a). By combining the organoid method with recently developed gene editing techniques such as CRISPR, researchers can now study developmental processes and disorders on an unprecedented level.

In this review, we will discuss the power of three-dimensional *in vitro* models with particular emphasis on the comparison to *in vivo* development, and how they can be used to model brain evolution and neurodevelopmental disorders. We will also discuss the near and far future directions, and how further methodological improvements could impact the field of neurogenesis research and development of new therapies.

## 2 Establishment of *in vitro* cultures

The first instances of explanting pieces of an organism and culturing these grafts to monitor the behavior of cells took place 110 years ago, with the seminal work of Harrison in which he observed neurons growing out from a frog embryo graft cultured in, what would later become known as, *in vitro* conditions (Harrison, 1906). This is considered the beginning of the tissue culture era. Numerous researchers since then have contributed to the elaboration and refinement of *in vitro* culture, producing methods to maintain whole tissue grafts for prolonged periods of time, thus enabling detailed studies of various physiological and cell-biological processes taking place. The establishment of the first immortal cell lines (Earle et al., 1943, Gey and Coffman, 1952) removed the obstacle of repeated tissue acquisition, and paved the way for a whole new field of cell-biological research.

In the field of neuroscience, methods of culturing adult and embryonic neurons has allowed for careful characterization of diverse neuronal processes, such as migration from an explant (Lapham and Markesbery, 1971) and identification of axon guidance cues (Kennedy et al., 1994). Although very useful for the study of neuronal physiology, these cultures can be problematic because they do not proliferate, and their maintenance is laborious and very limiting (Hydén, 1959). The establishment of a clonal neuronal line derived from a mouse neuroblastoma (Augusti-Tocco and Sato, 1969, Schubert et al., 1969) allowed for a more controlled production of neurons, thus eliminating the need for repeated explants. However, these cultures were still hindered by an inability to accurately model developmental events such as timed neurogenesis.

While two-dimensional cell cultures have provided important insight into cell biology, these often homogeneous cultures have unfortunately been lacking when it comes to understanding tissue development. Animal tissues are composed of a broad repertoire of cell types organized in complex 3D arrangements, which influence both cell identity and function. This organization cannot be recapitulated using traditional *in vitro* cell culture approaches. For example, even when cells were taken from the embryonic or adult forebrain, and were demonstrated to be intrinsically capable of recapitulating the main events of brain development (differentiation into neurons and glia) (Carpenter et al., 1999, Vescovi et al., 1999), and even formation of 3D structures called neurospheres (Reynolds and Weiss, 1992), they were still incapable of modeling the developing brain accurately. Therefore, in order to more accurately model the development of the tissue, a new approach had to be adopted, one that would model both the proper 3D organization and the development of the whole repertoire of tissue cell types.

A breakthrough in the form of establishment of embryonic stem (ES) cell lines (Evans and Kaufman, 1981, Martin, 1981, Thompson et al., 1989), which can be directed into producing cells of any germ layer, has allowed for the development of *in vitro* cultures that more accurately model cell fate conversions during embryonic development. Then, ten years ago, another leap forward took place, with the introduction of induced pluripotency (Park et al., 2008, Takahashi et al., 2007, Yu et al., 2007). Induced pluripotent stem (iPS) cells allow for the formation of a stem cell line from almost any type of tissue, and consequently present a highly valuable tool in basic and clinical research as they can be derived from living human patients.

Since the establishment of these pluripotent stem cell lines, various approaches have been devised for differentiation of a range of neural identities. By using a defined set of signaling molecules, almost any tissue could be produced *in vitro*, including neurons from the central and peripheral nervous system. For example, spinal motor neurons can be produced by a sequence of steps which caudalizes and ventralizes the ES cells by using retinoic acid and sonic hedgehog, respectively, thus recapitulating the *in vivo* signaling cascade (Wichterle et al., 2002). Using a controlled protocol of administering supplements, a more faithful developmental sequence of events could be achieved. However, as we will see, the human brain possesses an exquisite developmental complexity, and replicating it *in vitro* has been a huge challenge. Nonetheless, recent advances using 3D self-organizing cultures are beginning to provide better models of key neurodevelopmental events.

## 3 The complexity of the developing human brain

The human brain contains billions of neurons and glial cells, which form an elaborate and determined pattern of circuitry in the adult individual. Such complexity is reflected in its development, which takes place throughout most of embryonic and fetal development (Malik et al., 2013), and is composed of an intricate sequence of changes in the progenitor population with different proliferation potentials. Although a lot of progress has been made in recent years concerning our knowledge of non-human species, we will focus here on the development of the human brain. The human brain, although unremarkable from a gross morphological point of view, represents one of the most complex, if not the most complex organ, produced during embryonic development in the whole of the animal kingdom. The human brain is highly enlarged, compared to our closest relatives and ancestors, develops slower and for a longer time, and has a very complex configuration, with more types of neurons and brain areas, than in other mammalian lineages (Geschwind and Rakic, 2013).

**Fig. 1:**
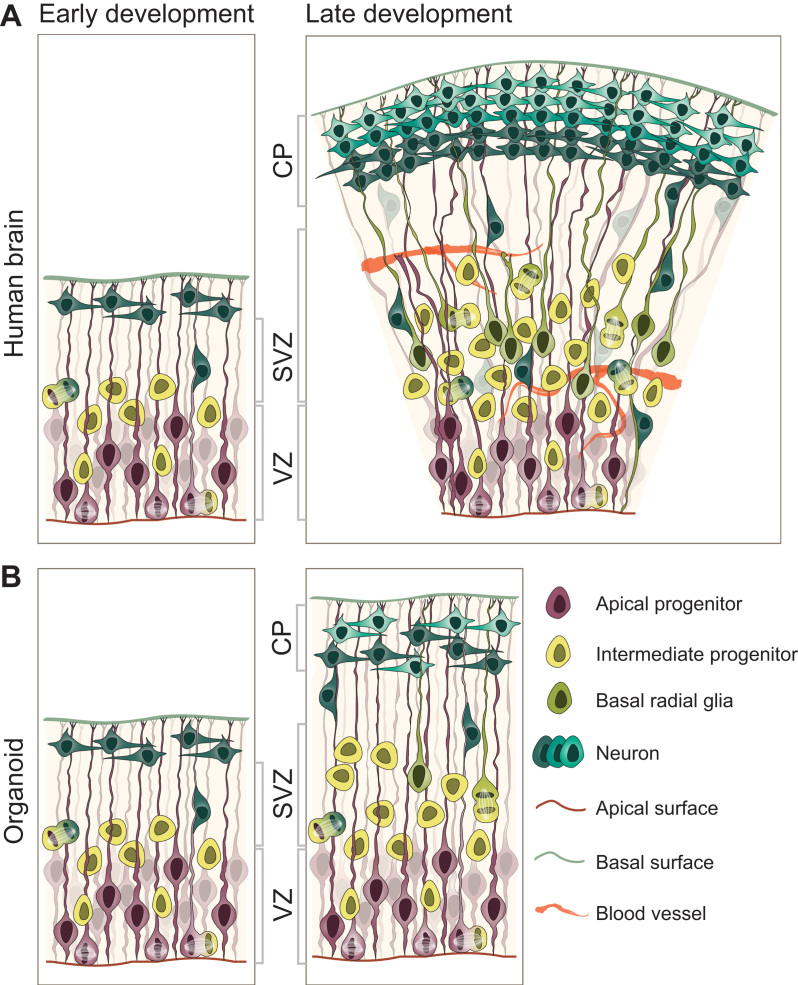
Comparison of *in vivo* and *in vitro* brain development. A simplified representation of the cell biological complexity of the *in vivo* developing brain and the *in vitro* brain organoid. The early stages (left) possess a similar morphological level of complexity. Later stages (right) differ in the size of the cortical wall and diversity and complexity of neural progenitor populations. Note the absence of vasculature (orange) in the organoid, a reduced SVZ and the rudimentary organization of the neuronal layers. VZ – ventricular zone, SVZ – subventricular zone, CP – cortical plate.

The human brain starts its development from a closed sheet of epithelium, the neural tube, populated by precursors of all other progenitor populations called neuroepithelial (NE) cells (Götz and Huttner, 2005). The neural tube encloses a fluid-filled cavity that later develops into brain ventricles. These elongated cells make up the whole of the neuroepithelium at early stages of brain development (before any neurogenesis commences) and divide by proliferative divisions in order to increase the number of neural progenitors, thus expanding the neuroepithelium laterally. As the layer consisting of NE cells is located right next to the ventricle, it is termed the ventricular zone (VZ) (Fig. 1A). As in most developing epithelia, mitoses take place at the apical surface, next to the ventricle, and in order to reach the right position for cell division, nuclei of NE cells must undergo a directed movement towards the apical side of the neuroepithelium, termed interkinetic nuclear migration (INM), a process which lasts during the whole period of neurogenesis (Taverna and Huttner, 2010). Just before the onset of neurogenesis, *i.e.* before any neurons are born, NE cells change some of their cell biological features and transform into radial glia (RG) (Götz and Huttner, 2005). RG switch their division mode from symmetric, proliferative, to asymmetric, self-renewing division (in which the progeny consists of one radial glia and one neuron or a different type of progenitor, see below) (Lancaster and Knoblich, 2012), although they still maintain vertical division planes (Kosodo et al., 2004).

The moment of first birth of neurons marks the onset of neurogenesis, and in human development this happens relatively early, at 5–6 gestational weeks (GW) (Howard et al., 2008). In addition to these first neurons, RG start producing another type of progenitor cell, which will lose the epithelial features and delaminate from the VZ. As they migrate towards the basal side of the neuroepithelium, they will establish another progenitor layer termed the subventricular zone (SVZ) (Fig. 1A), which can be distinguished from the VZ based on the orientation of the cells, and on the expression of some molecular markers (Fish et al., 2008, Smart et al., 2002). These progenitors mostly divide symmetrically to produce two neurons, and, because they represent an intermediate state between RG and neurons, are termed intermediate progenitors (IPs) (Haubensak et al., 2004, Miyata et al., 2004, Noctor et al., 2004). In addition to IPs, RG produce another type of progenitor, the basal (bRG) or outer radial glia (oRG) (Fietz et al., 2010, Hansen et al., 2010, Reillo et al., 2011) (Fig. 1A). bRG also settle themselves in the SVZ, which in the human is enlarged, as compared to other species (Smart et al., 2002). These cells then undergo asymmetric, self-renewing divisions to continue producing neurons but also replenish themselves. Although previously thought to be clearly defined and separate subpopulations of neural progenitors, it appears that IPs and bRGs (now collectively termed basal progenitors (BPs)) can fluctuate morphologically and in terms of their proliferative potential (Betizeau et al., 2013). It is thought that the complexity of the SVZ compartment and the ability of neural progenitors to remain or switch to a “more proliferative” state, coupled with the length of neurogenesis, may have contributed to evolutionary expansion of the neocortex. This expansion is noticeable in several mammalian lineages, but the human lineage especially stands out in this regard (Lewitus et al., 2014). In the end, all types of neural progenitors give rise to neurons, which migrate basally, using the RG processes. They settle themselves in well-organized layers, which have an inside-out pattern (the oldest-born neurons make up the deepest layers), finally making up the mature, six-layered cortical plate of an adult neocortex.

## 4 Modeling human brain developmental complexity *in vitro*

Even this very simplified description of human neocortical neurogenesis (for details of cell-biological features of neural progenitors, please see recent reviews (de Juan Romero and Borrell, 2015, Fernández et al., 2016, Florio and Huttner, 2014, Lui et al., 2011, Paridaen and Huttner, 2014, Taverna et al., 2014)) hopefully gives some indication of the complexity of cell biological and morphological changes that need to take place in order for a mature and functional adult brain to develop. Therefore it is remarkable that some *in vitro* protocols have managed to faithfully recapitulate a significant portion of the neurogenic period. The first protocols that mimicked some of the morphological aspect of the developing neocortex were 2D cultures, in which human ES or iPS cells were directed into cortical precursors by using different signaling molecules. The most prominent morphological signature of these systems was the organization of cells into neural rosettes, *i.e.* cells surrounding a central lumen and undergoing mitoses at the luminal side (Chambers et al., 2009, Elkabetz et al., 2008; Zhang et al., 2001). This organization is highly reminiscent of the organization *in vivo* in which NE cells and RG are organized radially around a fluid-filled ventricle. Furthermore, neural progenitors exhibited temporally controlled neuronal production, as evidenced by the successive emergence of molecular markers characteristic of different neuronal layers (Chambers et al., 2009, Elkabetz et al., 2008, Gaspard et al., 2008). This speaks to an intrinsic mechanism of *in vitro* derived neural progenitor cells, which are able to recapitulate the major milestones in the production of neocortical neurons. Subsequent improvement of this 2D method saw an increase in the complexity of the composition of neural rosettes, together with improved morphological similarities, like well-established INM (Shi et al., 2012). This method also produced a more complex configuration of neural progenitors, including bRG-like cells, with the typical morphology and molecular signature (PAX6 expression). An important quality of these methods is that they give rise to layer-specific neurons, which are capable of producing action potentials (Espuny-Camacho et al., 2013, Kirwan et al., 2015, Shi et al., 2012), develop synapses (Shi et al., 2012) and, when grafted into a cortex of mice, can integrate successfully among cortical neurons in a layer-specific manner (Espuny-Camacho et al., 2013, Gaspard et al., 2008).

A crucial advance in approaching an *in vitro* model more similar to the *in vivo* developing brain was made with the introduction of 3D culture methods (Eiraku et al., 2008, Watanabe et al., 2005), and further improvements of this serum-free culture of embryoid body-like aggregates (SFEB) (Mariani et al., 2012, Paşca et al., 2015). Here, in addition to characteristics already achieved by 2D cultures (INM, different progenitor populations, sequential birth of neurons characteristic of different layers), the cultures showed a well-defined ventricle, with a clearly distinguishable VZ and SVZ (Fig. 1B). Further studies combined the floating 3D aggregate approach with components of extracellular matrix (ECM), either by dissolving it in the medium (Kadoshima et al., 2013, Nasu et al., 2012), replating the free-floating cultures onto ECM-coated dishes (Mariani et al., 2012), or by embedding the growing embryoid bodies into pure Matrigel (Lancaster et al., 2013). These studies demonstrated the importance of the ECM as a crucial cue for proper organization of the neuroepithelium *in vitro* (Nasu et al., 2012). ECM components improved the polarization of the neural progenitor sheets and supported the development of elongated neuroepithelia, which surrounded lumina resembling the *in vivo* ventricles, and not just rosette formations. A significant improvement was also the appearance of neuronal “layers”, in which earlier born neurons, as labeled by deep-layer markers, were situated below later born neurons, as in the developing human brain (Kadoshima et al., 2013, Lancaster et al., 2013, Nasu et al., 2012). This layering is rudimentary (Fig. 1B, right – note the irregular arrangement of neurons, as compared to the *in vivo* brain), and does not persist throughout the late stages of the culture, after all of the neuronal layers have been born. A significant improvement was presented in a recent study (Qian et al., 2016), which showed the generation of neurons corresponding to all six layers. We are confident that further developments will build on these protocols and phenocopy other human developing brain characteristics (*e.g.* preplate splitting).

We have so far neglected culture methods that support the development of parts of the brain other than the neocortex. The majority of methods described to date have established approaches for the selective generation of particular brain regions (pituitary (Suga et al., 2011), hypothalamus (Wataya et al., 2008), cerebellum (Muguruma et al., 2015, Muguruma et al., 2010), retina (Eiraku et al., 2011)) and/or for the specific promotion of the telencephalic (forebrain) fate, by the addition of patterning factors. An alternative approach using combined Matrigel embedding and free floating conditions that bypasses the need for using signaling molecules instead leads to the generation of a variety of brain regional identities (Lancaster et al., 2013). Because of the presence of broad regional identities, this method was termed cerebral organoids, or brain organoids. In turn, it allows for self-patterning and self-organization processes to take place, resulting in distinct and interdependent brain regions appearing in the same organoid. These discrete brain regions are not randomly dispersed around the organoid, but show some patterns of regionalization as in the early developing brain, *e.g.* establishment of midbrain/hindbrain boundary with appropriate regions located adjacent to each other. Additionally, organoids exhibit some of the features of regional connections, with interneurons, which *in vivo* are partly produced in the ventral forebrain and migrate to the dorsal cortex (Nakajima, 2012, Wonders and Anderson, 2006), showing a similar pattern of migration in the organoids. It will be interesting to further study these potential communication pathways in the whole brain cultures, and whether the cues driving these migration trails correspond to the ones observed *in vivo.*

On a cellular level, organoids show a high level of similarity to the *in vivo* developing human brain in the early stages of development. The progenitor zones (VZ and SVZ) are easily recognizable, as in previously described methods, but the zones show a higher degree of complexity and sub-compartmentalization into an inner- and outer SVZ, which are separated by a neuronal fiber layer (Lancaster et al., 2013, Smart et al., 2002). The bRG also appear relatively frequently, although not as often as in the developing human brain (Fig. 1B). Due to the method limitations (lack of vascularization), the organoids are not able to mimic later stages of neurogenesis (see below).

**Fig. 2:**
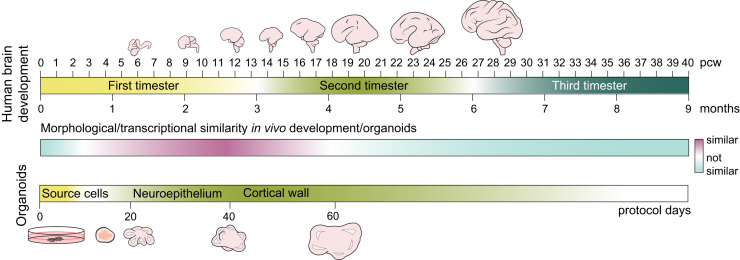
Timeline of human brain development. A timeline showing relative similarities between human *in vivo* brain development and the brain organoid protocol timeline. The relative similarity (cyan-purple gradient) is based on cell-biological and transcriptomics data from several studies and is not a quantitative measure. Human developing brain and brain organoids are not to scale. Abbreviations: pcw – post-conception weeks.

The previously mentioned methods of neocortical cultures show varying degrees of similarity to the developing human brain *in vivo*. However, human neocortical development is, as any other developmental process, comprised not only of obvious cellular differences in morphology and the small palette of markers that we have access to, but has to employ coordinated waves of gene expression in order to induce all of the morphological changes and patterning cues needed to develop a functioning brain. In order to determine the similarities of the *in vitro* methods to *in vivo* brain development, several studies have recently employed gene expression analyses on neocortical cultures by microarrays (Mariani et al., 2012, Paşca et al., 2015), RNA-seq (Mariani et al., 2015, Qian et al., 2016, van de Leemput et al., 2014) or single-cell RNA-seq (Camp et al., 2015), and compared it to gene expression of the developing brain (Camp et al., 2015, Kang et al., 2011, Miller et al., 2014). These studies all reported that *in vitro* methods replicate remarkably well early *in vivo* brain development (middle and end of the 8–10 gestation weeks (GW)), with some reports that the *in vitro* development parallels the *in vivo* up to late mid-fetal period (19–24 GW) (Paşca et al., 2015, Qian et al., 2016) (Fig. 2). All of the methods showed the correct employment of the frontal brain neurogenic program, confirming cell-biological analyses. A detailed, single-cell transcriptome analysis corroborated the cell-biological analyses and found that the *in vitro* methods produce relatively less BPs, as compared to the *in vivo* neocortical development (Camp et al., 2015). These differences are an obvious consequence of the methods’ limitations – the inability to vascularize the cultures and the possible lack of some intrinsic and extrinsic cues, which are not replicated with the current protocols. However, when transcriptomes of individual organoid cells were compared to the transcriptomes of the cells of the same lineage from an *in vivo* brain, the main differences did not come from the method of origin (*in vitro versus in vivo*), but from the state of cells themselves (AP, BP, neuron). The differences in the transcriptomes between the organoid and the *in vivo* cells that this study picked up, are mostly in the genes which have a low expression in the fetal brain, or in the genes that reflect the composition of the *in vitro* media culture and the responses to the factors present therein (Camp et al., 2015).

Taken together, the cell-biological, temporal and gene expression similarities between *in vitro* neocortical cultures and *in vivo* developing brain show that we are currently able to model early human neocortical development accurately. This opens the door to studies of human-specific brain disorders and basic biological mechanisms of development. The possible uses of these systems are boundless and have the potential to overcome the frequently observed lack of translation from animal studies. In addition, there is a further benefit with regard to ethical considerations of using animals where there is the potential to limit the numbers of animals needed for neurodevelopmental studies.

## 5 Modeling neurodevelopmental disorders *in vitro*

The faithful recapitulation of brain developmental processes is a relatively new event, so it does not surprise that studies using these 3D methods are still few. However, the field of developmental organoid research is currently booming (Fatehullah et al., 2016, Huch and Koo, 2015, Passier et al., 2016, Suzuki and Vanderhaeghen, 2015, Yin et al., 2016) and we can expect a surge of studies in the near future.

**Fig. 3:**
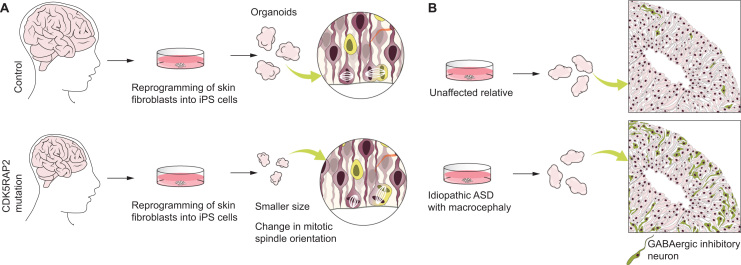
Brain organoids as a tool to study neurodevelopmental disorders. (A) Lancaster et al., 2013. used brain organoids to study brain development in a microcephalic patient with a mutation in CDK5RAP2 gene, which causes microcephaly. Organoids produced from the patient were smaller than the control, and had a change in the cleavage plane orientation of the APs, which might contribute to the decreased progenitor pool, and thus to the smaller brain size. (B) Mariani et al., 2015. used brain organoids to study idiopathic autism. Organoids generated from patients showed an increase in GABAergic inhibitory neurons (depicted in green), as compared to the healthy relatives.

The study that first described the protocol for growing growth factor-free organoids also used them to study a human neurodevelopmental disorder, primary microcephaly (Lancaster et al., 2013, Woods et al., 2005) (Fig. 3A). Truncating mutations in the CDK5RAP2 gene influence the neocortical progenitor pool and result in an overall smaller brain (Bond et al., 2005, Buchman et al., 2010). However, previous studies were conducted with mouse models, which did not recapitulate the symptoms of microcephaly at the same level as in humans. Fibroblasts from a severe microcephaly patient with CDK5RAP2 truncating mutations were used to produce iPS cells and grow organoids. These organoids already showed a crude phenotype, by being smaller than wild-type brain organoids. Analysis of neural progenitors in patient-derived organoids showed that they had an increased proportion of mitotic divisional planes that were not perpendicular to the ventricular surface. This is in contrast to the wild-type situation, in which the maintenance of the division plane perpendicular to the ventricular surface is crucial for the upkeep of the progenitor pool and for the proper balance between proliferation and neurogenesis (Lancaster and Knoblich, 2012) (Fig. 3A, right). The change in mitotic plane orientation might influence this sensitive balance and cause premature neuronal differentiation, by a mechanism similar to the one observed in the mouse model (Buchman et al., 2010). Microcephaly is coming into focus again, this time not only in the scientific community, but also in the general public, due to the current epidemic of the Zika virus, and its potential link to an increased number of infants born with severe microcephaly (Heymann et al., 2016). In this context, brain organoids have been a powerful tool for the rapid analysis of the effects of Zika on human brain development, providing insight in an extremely short time period. Several very recent studies reported an effect of Zika on neural stem cells (Tang et al., 2016) and on brain organoids (Cugola et al., 2016, Dang et al., 2016, Garcez et al., 2016, Qian et al., 2016). For example, the report by Qian et al. (2016) which used pure forebrain organoids, showed that the early exposure to two different strains of Zika virus influenced the proliferation of cells in the VZ and caused a phenotype resembling microcephaly observed in children prenatally exposed to the virus. Furthermore, although not directly analyzing the mechanism of Zika virus infection, Nowakowski et al. (2016) used brain organoids to identify a possible point of entry of the virus into neural progenitors. Finally, Dang et al. (2016) reported activation of innate immune signaling in cerebral organoids infected with Zika, suggesting a potential mechanism for apoptosis in neural progenitors.

Another neurodevelopmental disorder recently studied in organoids is autism spectrum disorder (ASD) (Geschwind, 2009) (Fig. 3B). Mariani et al. (2015) used iPS cell-derived organoids from patients with idiopathic ASD to study the processes taking place during neocortical neurogenesis that may contribute to the described complex pathologies. ASD-derived organoids, and their comparison to wild type organoids, showed that in the patients the early neural progenitors had a decreased cell cycle length, resulting in their over-proliferation. An additional feature of ASD organoids was that the production of GABAergic neurons was increased, due to the increase in expression of FOXG1, a gene involved in the production of early cortical neurons and in some ASDs with prenatal microcephaly (Hanashima et al., 2004, Jacob et al., 2009) (Fig. 3B, right). The ASD organoids also exhibited overgrowth of neurites and an increase in the number of synapses, which is one of the characteristics found in some *post-mortem* studies of ASD patients (Hutsler and Zhang, 2010). This study showed that the prenatal alterations in the proliferation/neurogenesis equilibrium could be one of the main features of at least a subset of ASDs, and it creates opportunities for focused prenatal diagnostics and drugs that might suppress the abnormal phenotype and/or alleviate the symptoms.

Schizophrenia represents another debilitating disorder, origins of which are thought to come partly from a disruption of neurodevelopment (Rapoport et al., 2012). Some regions of the genome show particular association with an increased risk of schizophrenia (Malhotra and Sebat, 2012), but the underlying mechanisms remain elusive. Yoon et al. (2014) used iPS cells-derived from patients with a deletion in one of the regions implicated in increased schizophrenia risk (15q11.2) to derive neural rosettes which model the behavior of early cortical neural progenitors. The authors noticed that, in the patient derived rosettes, the APs had a disrupted apical region, as labeled by the apical polarity markers. Although preliminary, the study captures the cellular phenotype, which follows from a particular aberrant genomic rearrangement, implicated in several neurological disorders. Further studies will hopefully delve deeper into the etiology of these complex disorders, and brain organoids will likely provide an important tool in these studies.

From a handful of studies that used brain organoids to study neurodevelopmental disorders, it is already apparent that these methods hold immense opportunities for studying human-specific disorders, which cannot be adequately modeled in the mouse (*e.g.* microcephaly, schizophrenia, autism). It is important to stress that, although the organoid method mimics human brain development remarkably well, there are many limitations, which hamper their use for the study of certain neuropathologies.

## 6 Limitations of the current methods

Understandably, *in vitro* organoid culture takes place without the normally present embryonic surrounding. This allows for the visualization of processes taking place in the tissue and manipulation of organogenesis, but it also means that the organoid tissue lacks essential developmental and patterning cues, which are necessary for development into a fully formed, mature organ. For brain organoids specifically, the lack of body axes mean that, although the organoids develop discrete brain regions, they do not organize themselves in the same pattern as present *in vivo* (Lancaster et al., 2013). Furthermore, the organoid method still suffers from the “batch syndrome”, in which different batches of organoids show significant variability in quality and brain regions they produce (Lancaster and Knoblich, 2014b). A recent development in this direction is the establishment of pure forebrain organoids, which, by changing the protocol conditions, can be directed into producing other region-specific organoids (Qian et al., 2016). Further improvements of the method, together with technical developments should be aimed at producing homogeneous, correctly patterned organoids.

A further limiting factor in the *in vitro* culture is the lack of vascularization. Early development of the neocortex progresses without vasculature, before blood vessels invade the cortical wall (Vasudevan et al., 2008). Late development, however, is highly dependent on the vascularization of the SVZ (Fig. 1B, right), as the proximity of the blood vessels represents a niche for neural progenitors (Javaherian and Kriegstein, 2009) and is necessary for efficient neural progenitor differentiation (Lange et al., 2016). This lack of vascularization is probably one of the factors influencing the scarcity of the SVZ progenitors and might also be partly responsible for the difficulties researchers have encountered in trying to replicate correct cortical plate formation. In order to faithfully model *in vivo* development, with all of its progenitor complexity, it is necessary to focus the efforts on delivering signaling molecules deep inside the tissue, either by means of cell culture modifications, or by engineering innovations. In addition, the problem of oxygen penetration renders the center of the organoid necrotic, which could interfere with its normal development, physiology and potential neuronal migration routes.

**Fig. 4:**
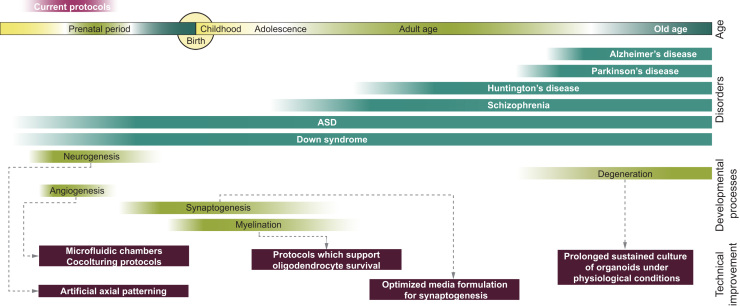
Potential future brain modeling capabilities. A simplified schematic representing human brain development timescale, indicating several milestones during embryonic development (green), which could be modeling in brain ogranoids in future, following certain technical improvements (dark purple, for details, please see main text) (for a more detailed timescale of milestones, please see (Silbereis et al., 2016)). The timescale also indicates the onset of a subset of neurodevelopmental and neurological diseases, which could be modeled (or partly already are) by using the brain organoid technique (petrol color).

Improvements in the organoid method will allow for a widening of the range of topics and problems that can be studied (Fig. 4). The ability of organoids to model later embryonic and fetal development will allow us to study the establishment of circuits and connectivity. Although cultures of neurons and organoids make functional synapses, we are still unable to maintain the cultures in conditions ideal for the establishment of proper circuitry. It appears that the current 3D organoid protocols favor progenitor cells, and the current media formulations do not support the establishment of mature synapses. Therefore it is necessary to adjust the conditions which, later in the organoid generation protocol, support neurophysiological activity (Bardy et al., 2015). This will be especially useful for further studies of schizophrenia and similar disorders, which are considered disorders of cortical connectivity, and a subset of them have a neurodevelopmental origin (Brennand et al., 2011, Rapoport et al., 2012).

In summary, on a cell-biological note, with current techniques, organoids present an excellent model for studying early human brain development, tissue morphogenesis, proliferation of neural progenitors, and their transformations from one cell state to another. Further refinements of the protocols will allow for the study of more complex interactions in the developing brain, like cell–cell (influences of progenitors and neurons on each other, synapses and connectivity) and cell-environment interactions (hypoxia, exposure to chemicals).

## 7 Brain organoids of the future

Brain organoids (and organoid systems in general), which adequately model tissue development and physiology, are a relatively new development, and the field has exploded in the last several years. Thus, it is easy to envisage that in 10–20 years from now (or even less) we will be able to almost fully mimic development of certain tissues *in vitro.* In addition, further improvements in the technique might allow us to model adult brain physiology and disorders of the adult and ageing brain.

The problem of heterogeneity of whole-brain organoids (see Chapter 5) might be solved by a combination of the accumulation of knowledge about stem cells and further technological improvements. New research about stem cells will bring about a deeper understanding of the starting material and the organoids produced thereof. As accumulating evidence shows that hES cells show significant variability which depends on the cell line (Rugg-Gunn et al., 2007), passage (Li et al., 2007), and even size of the colonies and the position of cells within the colony (Bauwens et al., 2008, Rosowski et al., 2015), careful choice of the cell line, method of reprogramming into iPS cells and handling of the cells have to be taken into account when developing new protocols. Correct axial patterning signals, which would in turn influence the predictable appearance of different brain regions could be delivered to the organoids by using signal-releasing beads, or by growing organoids on carriers coated with signaling molecules.

Some general protocols, which include combining tissue-specific cells with mesenchymal cells, to facilitate tissue vascularization upon transplantation of an organ bud (Takebe et al., 2015), could represent the foundations upon which novel organoid protocols with incorporated vascularization could be built. Another way to overcome the lack of vascularization could be a bioengeineering approach using microfluidic chambers (Bhatia and Ingber, 2014, van Duinen et al., 2015), which could drive the flow of fluid through the organoid and thus serve as vasculature. Growing organoids directly on a microfluidic chip (Giobbe et al., 2015), with the combination of signaling molecule carriers, to enable proper patterning, might be the way to go. In our efforts to develop vascularized organoids, we should also incorporate lessons from cancer research, as it is well established that tumors actively promote angiogenesis (Hanahan and Weinberg, 2011).

Improving the conditions for late stages of neurogenesis in the organoid culture might bring about the induction of myelination, which in humans is mostly a postnatal process, but it starts prenatally. Trisomy 21, or Down syndrome (DS) is one of the most common causes of intellectual disability (Hartley et al., 2015). It appears that the gene expression disequilibrium, caused by an additional copy of chromosome 21, has a profound effect on cortical neurogenesis, and especially on oligodendrocytes and the myelination process (Chakrabarti et al., 2010, Olmos-Serrano et al., 2016). Patient-derived brain organoids would allow for a detailed study of aberrant cortical development and the development of potential therapeutic strategies that would improve the myelination process in DS-sufferers. As the gene for amyloid precursor protein (APP), which is one of the main players in the development of Alzheimer's disease (AD), is on chromosome 21, people with DS have a markedly increased risk of developing this disorder, making DS organoids valuable also for modeling AD.

In the case of AD, most cases of the disease are late-onset, *i.e.* appear in individuals of 65 years of age and older. Currently we are using mouse models for both DS and AD, but, although very useful, they are inadequate to correctly model the etiology and progression of these complex human disorders (Hartley et al., 2015). Organoids of the future, which model the physiology of ageing neurons, might be able to provide further insight into cellular and molecular mechanisms of pathology and aid in developing drugs and treatments for the prevention and alleviation of disease symptoms. Interestingly, some DS sufferers do not develop AD (Wiseman et al., 2015), and these individuals and their derived organoids could prove instrumental in deciphering the genetic backgrounds than confer resistance to early-onset AD.

Functional vascularization of the organoid may prove to be the most challenging, but also the most rewarding aim of future improvements. The interaction of blood vessels and the brain tissue itself is a field with high potential for direct clinical applications. The delivery of drugs to brain tissue is limited by the blood-brain barrier, which restricts the admission of chemicals to neurons, so modeling it faithfully will allow us to improve the efficiency of drug application, and the physiology of drug transport through the blood-brain barrier. In addition, the blood-brain barrier is dysfunctional or compromised in many brain disorders (Abbott, 2013, Zhao et al., 2015, Zlokovic, 2011), and a vascularized brain organoid would be a powerful asset in fully comprehending the etiology of these neurological diseases. Furthermore, it appears that the disruption of the blood-brain barrier is a common feature of the aging process (Montagne et al., 2015), and this would represent an interesting avenue of discovery. Finally, vascularized organoids, which mimic adult brain physiology, could also be used as models for stroke and post-stroke recovery and present an ideal platform for drug testing.

There are many other diseases of the adult central nervous system that are excellent candidates to be studied in brain organoids. Just to name a few, Parkinson's disease, Huntington's disease, and various motor neuron diseases are debilitating disorders which affect an increasing number of individuals, as the average human life span rises. Of particular interest is Huntington's disease, a debilitating heritable condition, brought about by an increase in the number of glutamine repeats (polyQ) in the gene huntingtin (Brouwer et al., 2009; Li and Bonini, 2010). As the severity and the age of onset of the phenotype depends on the number of polyQ repeats, a combination of the organoid method and CRISPR gene editing technique might give us insight into the mechanisms by which these repeats cause the disease. The same approach could be applied to other polyQ neurological diseases.

**Fig. 5:**
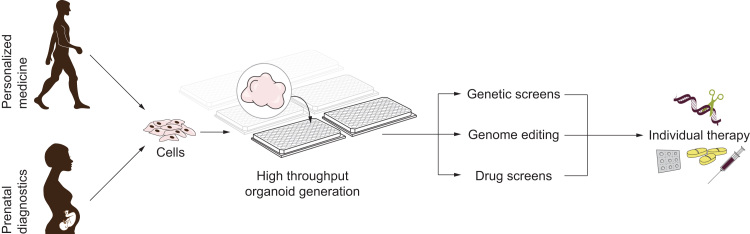
Personalized medicine using brain organoids. Brain organoids in the future might be used in a personalized medicine approach. Individual-derived cells (obtained as a part of a preventive monitoring or disease diagnostics) or cells derived from the embryo as a part of prenatal diagnostics can be reprogrammed into iPS cells, and brain organoids on a large scale could be generated. This large number of organoids could be used for genetic screens and drug screening. Genome editing could also be performed to test the outcome on the brain phenotype. With this in mind, personalized therapeutic strategies may be designed.

“Personalized organoids” – organoids directly derived from the affected person, together with drug testing, would represent an effective clinical usage of the organoid method (Brennand et al., 2015, Fatehullah et al., 2016, Wen et al., 2016) (Fig. 5). Personalized medicine would mean that clinicians and researchers would need to obtain cells from a patient, grow brain organoids on a high throughput scale and test the effectiveness of a large set of drugs, finding the ones most appropriate for the patient. A similar pipeline might be envisaged as a part of preventive medicine, or even prenatal diagnostics, as non-invasive methods of embryo genome analysis and cell acquisition are developed (Gregg et al., 2013). Both of these strategies call for the establishment of organoid production on a higher scale. With current methods, organoids can be produced in the hundreds, but in order to test a palette of drugs, one would need to produce thousands of organoids from a single individual. Automation of the process will be of crucial importance in this. An exciting new step forward has been made recently, with the development of a 3D-printed, scalable set of mini bioreactors named SpinΩ, which allows for paralleled production of a large number of organoids and under various conditions (Qian et al., 2016).

Although mostly discussed in the context of human brain development and human pathologies, brain organoids also provide a platform for evolutionary studies, enabling direct comparison between development of species, brains of which are not readily available (*e.g.* apes and primates) (Otani et al., 2016). As the field of comparative cell biology of neocortical neurogenesis recently experienced a *renaissance* (Fietz et al., 2010, García-Moreno and Molnár, 2015, García-Moreno et al., 2012, Kelava et al., 2012, Lewitus et al., 2014, Reillo et al., 2011), we are sure that the development of protocols for different mammalian species will deepen our insight into evolutionary aspects of neurogenesis.

Although some of the future ideas presented in this chapter are still science fiction, we believe that it is necessary to discuss potential long-term or even far-fetched goals, in order to inspire different ways of thinking and encourage tighter collaborations among basic researchers, clinicians and bioengineers. We are convinced that a multidisciplinary approach such as this will yield new insights and has the most potential to drive the field forward.

## 8 Conclusion

In this review we presented the current state of the field of brain organoid research, with special attention to how well the current protocols recapitulate human brain development. We have also introduced the current limitations of the technique, and what the logical next steps may be in the development of these methods. What is clear is that, although tremendous advances have been made in improving the *in vitro* culture of developing neural tissues, these methods are not without their faults and limitations. Improvements in the techniques will allow for more complex processes to be studied, including intricate cell-cell interactions and migration in the developing brain. Furthermore, diseases other than severe, early neurodevelopmental disorders could be modeled, with the potential to model more common, but also more subtle, disorders. With our outlook into the future, we would like to emphasize the vast potential of brain organoid research and inspire future methodological improvements.
